# Resonant Capacitive MEMS Coupled to a T-Shaped Acoustic Cavity for Enhanced Photoacoustic Gas Detection

**DOI:** 10.3390/s25247523

**Published:** 2025-12-11

**Authors:** Fanny Pages, Julien Charensol, Tarek Seoudi, Julie Goutorbe, Loni Laporte, Diba Ayache, Fadia Abou Naoum, Eric Rosenkrantz, Aurore Vicet, Michael Bahriz

**Affiliations:** Institut d’Électronique et des Systèmes (IES), Centre National de la Recherche Scientifique (CNRS), Univ Montpellier, 34095 Montpellier, France; fanny.pages1@umontpellier.fr (F.P.); julien.charensol@umontpellier.fr (J.C.); tarek.seoudi@umontpellier.fr (T.S.); julie.goutorbe1@umontpellier.fr (J.G.); loni.laporte@hotmail.com (L.L.); diba.ayache@umontpellier.fr (D.A.); fadia.abou-naoum@umontpellier.fr (F.A.N.); eric.rosenkrantz@umontpellier.fr (E.R.); aurore.vicet@umontpellier.fr (A.V.)

**Keywords:** photoacoustic spectroscopy, gas sensor, MEMS, capacitive transduction, acoustic cavity

## Abstract

To address the lack of compact and high-performance gas sensors in the literature, a miniaturized photoacoustic sensor has been developed using a resonant capacitive MEMS specifically designed for gas detection. Its performance is enhanced by coupling it to a T-shaped acoustic cavity, which confines and directs the acoustic waves toward the transducer. Electrical and photoacoustic characterizations were carried out to determine the nominal capacitance and resonance frequency of the device. The acoustic coupling resulted in a significant improvement in the transducer’s mechanical response, while the linearity of the sensor was confirmed over a broad concentration range. This improvement led to a reduction in the limit of detection (LOD) from 186 ppmv to 16 ppmv. In parallel, the Normalized Noise-Equivalent Absorption (NNEA) metric improved from $1.49\times 10^{-7}\;W\cdot {\mathrm{cm}}^{-1}\cdot {\mathrm{Hz}}^{-1/2}$ to $1.28\times 10^{-8}\;W\cdot {\mathrm{cm}}^{-1}\cdot {\mathrm{Hz}}^{-1/2}$, representing a 11-fold increase in sensitivity. Stability over time is confirmed through Allan–Werle deviation analysis, confirming the reliability of the signal over extended measurement periods. These results demonstrate that coupling a resonant MEMS transducer to a well-designed acoustic cavity is an efficient strategy to significantly improve the sensitivity of photoacoustic gas detection systems.

## 1. Introduction

Nowadays, detecting and measuring harmful gas concentrations is crucial to protect both the populations and the environment. This applies to industrial leak detection, civil protection, and medical diagnostics based on exhaled biomarkers. Such applications demand sensors with high sensitivity, meaning the ability to detect very low concentrations of specific gases, and high selectivity, referring to the capacity to discriminate the target gas from other components in the environment. Additionally, these sensors must be compact to ensure better portability and capable of real-time measurement. In the case of methane, safety considerations further highlight the need for reliable and sensitive detection. Methane is a highly flammable gas with well-defined explosion limits between 5% (lower explosive limit, LEL) and 15% (upper explosive limit, UEL) in air [1]. At high concentrations, methane can also displace oxygen, creating an additional asphyxiation risk. These hazards reinforce the need for compact, selective, and real-time methane sensors capable of detecting leaks early, ensuring industrial and environmental safety.

In recent years, several gas sensors have been developed to detect and quantify a wide range of gases. The most used gas sensors are electrochemical, semiconductor, and infrared sensors [2]. Electrochemical gas sensors operate by generating a current through a redox reaction involving the target gas, with the current magnitude directly related to the gas concentration. When considering methane detection, such sensors are widely used due to their low cost and sufficient selectivity, achieving detection limits as low as 9 ppmv [3] (10,000 ppmv corresponds to 1% *v*/*v*). Semiconductor-based gas sensors, on the other hand, rely on changes in the electrical conductivity of the sensing material induced by interactions between gas molecules and the sensor surface. These sensors are also low-cost and offer the advantage of high compactness, making them well suited for portable applications; they can achieve even lower detection limits, down to 5 ppmv for methane [4]. Among the main gas sensing technologies, infrared sensors using tunable diode laser spectroscopy (TDLS) are considered the most selective, sensitive, and reliable. For methane detection, they can reach detection limits in the parts-per-billion (ppb) range, as reported by [5]. This technique uses a modulated infrared laser whose emission line is much narrower than the absorption band of the target gas, allowing highly specific detection. The light that passes through the gas is measured by a photodiode. According the Beer Lambert law, the sensitivity depends on the optical path length which means that the sensor’s performance strongly depends on the size of the sensor, reducing its compactness. Photoacoustic spectroscopy (PAS) is a technique based on TDLS that replaces optical detection with acoustic detection. Unlike TDLS, the sensitivity of PAS only depends on the laser power, which enables the development of significantly smaller sensors. In PAS sensors, when the laser’s emission wavelength matches the gas absorption wavelength, the gas molecules absorb the energy and become excited. As they return to their fundamental state, they release this energy in the form of heat through non-radiative relaxation. This localized heating generates periodic pressure variations, producing acoustic waves at the same frequency as the laser modulation. The resulting acoustic waves are then collected by a detector, whose type depends on the specific photoacoustic technique used. Three main approaches are commonly employed:

- Microphone-based photoacoustic spectroscopy (MPAS) uses capacitive microphones for signal transduction. Although compact and easy to integrate, microphones are highly sensitive to ambient noise due to their flat frequency response, which makes it difficult to suppress background fluctuations.
- Cantilever-enhanced photoacoustic spectroscopy (CEPAS) relies on a silicon cantilever combined with optical readout. The silicon cantilever, while offering high sensitivity, is not well suited for miniaturized systems because of its bulky optical detection setup.
- Quartz-enhanced photoacoustic spectroscopy (QEPAS) employs a quartz tuning fork (QTF) with piezoelectric transduction. The quartz tuning fork, despite its high quality factor, was not originally designed and optimized for photoacoustic sensing.

In response to the limitations of these existing methods, we have developed, since 2019 [6], a new approach called MEMSPAS (MicroElectroMechanical Systems PAS). This technique is based on a a silicon capacitive resonator fabricated from a silicon-on-insulator (SOI) substrate combining the high quality factor of the quartz tuning fork with the sensitivity of the silicon cantilever, while being specifically designed for photoacoustic detection. The latest prototype surpassed the state of the art, but further improvements in performance were actively pursued [7]. The present work aims to further enhance the sensitivity of MEMSPAS by coupling the MEMS transducer to a dedicated acoustic cavity. To this end, a T-shaped acoustic cavity was designed to match the resonance frequency of the MEMS. In this configuration, a standing acoustic wave is generated within a rigid tube structure, which confines and directs the acoustic energy toward the resonator, thereby amplifying its mechanical response. According to the literature, such acoustic coupling can theoretically provide a sensitivity gain of up to a factor of 30 [8,9] when resonance conditions are met. This study focuses on the experimental characterization of the sensor both with and without the acoustic cavity, to quantify the resulting performance enhancement. Key performance indicators, including the limit of detection (LOD) and the normalized noise-equivalent absorption (NNEA) metric, are used to assess the effectiveness of the acoustic coupling strategy and evaluate the influence of the cavity design on the overall sensitivity of the system.

## 2. Design

### 2.1. Silicon Micro-Mechanical Resonator Design

**Figure 1:**
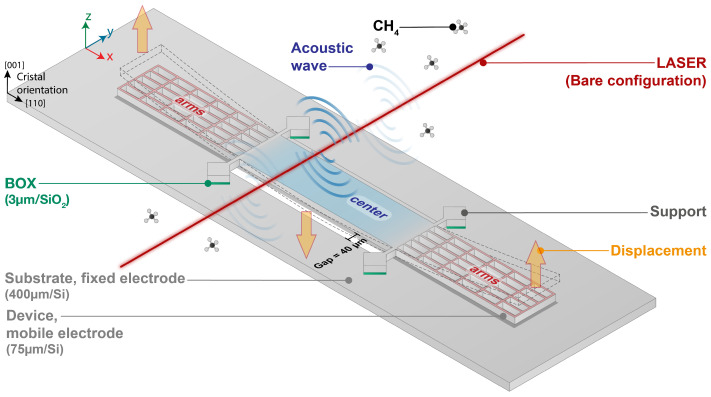
Schematic illustration of the H-square resonator deflexion under photoacoustic excitation. The resonator consists of two main parts: a central region, which collects the photoacoustic energy, and surrounding arms for capacitive transduction. The modulated laser beam is focused on the center of the resonator and oriented perpendicularly to its optical axis.

The mechanical resonator used in this study, referred to as the H-squared resonator, is specifically designed for photoacoustic applications and is illustrated in Figure 1. Its geometry was optimized in 2023 to enhance photoacoustic energy collection and maximize the final signal-to-noise ratio (SNR) of the sensor [7]. The H-squared resonator has overall dimensions of 8530 µm in length, 805 µm in width, and 75 µm in thickness. Notable geometric features include a back opening and twelve thin arms, each 13 µm wide, which help to minimize viscous damping and improve acoustic sensitivity. The resonator is centered above the back opening, with a clearance of 40 µm on each side between its edges and those of the opening. The effective mass of the structure is estimated at $4.41\times 10^{-8}$ kg. The MEMS device is fabricated on a double-side polished (100)-oriented silicon-on-insulator (SOI) wafer using the fabrication process detailed in [10]. The SOI structure is composed of a 75 µm thick device layer, a 3 µm buried oxide (BOX) layer, and a 400 µm handle layer. Both the device and handle silicon layers are heavily boron-doped, giving a resistivity of 0.01∼0.02 Ω· cm. This high doping level eliminates the need for metal electrodes by enabling capacitive transduction directly through the silicon. As shown by Figure 1, during operation, a wavelength-modulated laser is focused above the center of the resonator. When the laser’s wavelength matches the absorption line of the target gas, the light is absorbed, and the photoacoustic effect generates localized acoustic waves along the modulated laser beam. The pressure fluctuations generated by the acoustic waves cause the central part of the resonator to oscillate, which in turn actuates the surrounding arms in a vertical and in-phase motion with each other but in antiphase with the central part. In this vibration mode, when the center moves upward, the arms move downward, and vice versa. The motion of the arms is higher than that of the center one and leads to a large gap variation between the arms (movable electrode) and the substrate (fixed electrode), producing a measurable capacitive signal. This efficient conversion of acoustic energy into an electrical signal makes the H-squared resonator well suited for highly sensitive photoacoustic gas detection.

### 2.2. Acoustic Cavity

**Figure 2:**
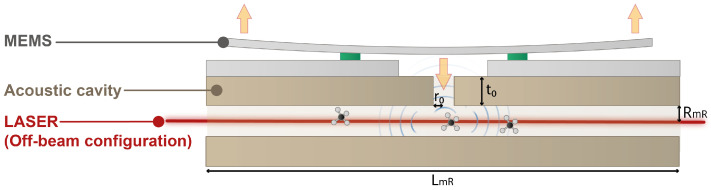
Schematic illustration of a T-shaped acoustic cavity. A modulated laser beam passes through the main tube, with length $L_{\mathrm{mR}}$ and radius $R_{\mathrm{mR}}$, where acoustic waves are generated via the photoacoustic effect. These waves are then guided through a branch tube, with length $t_{0}$ and radius $r_{0}$, toward the MEMS. The resonance frequency of the acoustic cavity mainly depends on the length $L_{\mathrm{mR}}$ of the main tube.

The previously described MEMS resonator is then coupled to a T-shaped acoustic cavity. The acoustic cavity is placed directly below the MEMS, and a modulated laser beam passes through a main horizontal tube where acoustic waves created by photoacoustic effect are confined, as shown in Figure 2. The waves are then guided via a perpendicular branch tube toward the MEMS.

For optimal photoacoustic signal detection, the resonance frequencies of the MEMS and the acoustic cavity $f_{AC}$ must match. This matching is achieved by adjusting the length $L_{\mathrm{mR}}$ of the main tube, based on the combination of the following equations proposed in [9]:

(1) $$L_{\mathrm{mR}}=L_{\mathrm{eff}}-2C_{\mathrm{mR}}\cdot R_{\mathrm{mR}}$$

(2) $$L_{\mathrm{eff}}=\frac{c_{s}}{f_{AC}}-\frac{c_{s}}{\pi f_{AC}}\tan^{-1}\left(4\pi \frac{R_{\mathrm{mR}}^{2}}{r_{0}^{2}}\cdot \frac{t_{\mathrm{eff}}f_{AC}}{c_{s}}\right)$$

(3) $$t_{\mathrm{eff}}=t_{0}+C_{t0}\cdot r_{0}$$

**Figure 3:**
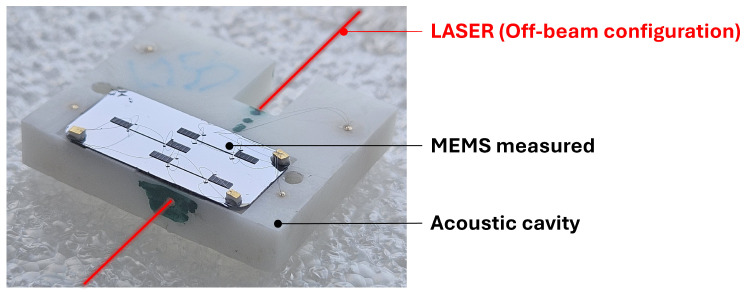
Image showing the H-squared resonator above the acoustic cavity.

Here, $c_{s}$ is the speed of sound in air (343 m/s), and $L_{\mathrm{eff}}$ and $t_{\mathrm{eff}}$ are effective lengths that take into account the acoustic wave radiation at the tube openings as described in [9]. $C_{\mathrm{mR}}$ and $C_{t0}$ are corrective coefficients [11]. The chosen acoustic cavity is fabricated using stereolithography (SLA/3D printing). The MEMS is then mounted above the cavity and electrically connected using aluminium wires to measure the capacitive output current. The final assembly is illustrated in Figure 3.

## 3. Electrical Measurements

**Figure 4:**
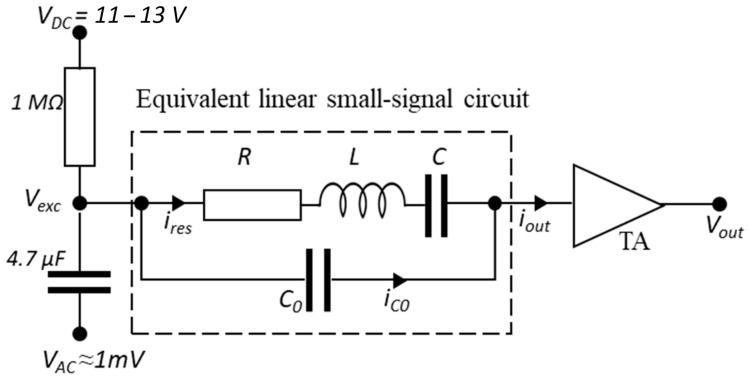
Electrical characterization circuit. $V_{\mathrm{DC}}$, $V_{\mathrm{AC}}$, $V_{\mathrm{exc}}$, and $V_{\mathrm{out}}$ are the polarization voltage, drive voltage, excitation voltage, and output voltage, respectively. The output current $i_{\mathrm{out}}$ is composed of two components: $i_{\mathrm{res}}$ and $i_{C_{0}}$, which are the currents flowing through the RLC branch and the $C_{0}$ branch, respectively.

The goal of electrical characterization is to determine the resonance frequency, the quality factor, and the nominal capacitance of the device. The circuit used for this characterization is shown in Figure 4. The resonator is equivalent to a linear small-signal circuit consisting of a series RLC circuit in parallel with a capacitance $C_{0}$, according to the Butterworth-Van Dyke model [12]. The capacitance $C_{0}$ represents the equivalent static (non-vibrating) capacitance of the MEMS device [7].

At the input of the resonator, a low-pass filter composed of a 1 MΩ resistor and a 4.7 μF capacitor is added to protect the circuit, improve stability, and reduce high-frequency noise. A DC bias voltage ($V_{\mathrm{DC}}$) and an AC drive signal ($V_{\mathrm{AC}}$) are applied to the input. This induces the motion of the MEMS structure via electrostatic forces between the fixed substrate and the movable arms, leading to a capacitance variation. The resulting output current $i_{out}$ is then amplified and converted to voltage using a transimpedance amplifier (TA) with a $10^{8}$ gain ($G_{\mathrm{TA}}$), placed at the output of the circuit. The resulting signal is processed by a Zurich Instruments MFLI lock-in amplifier (Zurich, Switzerland).

**Figure 5:**
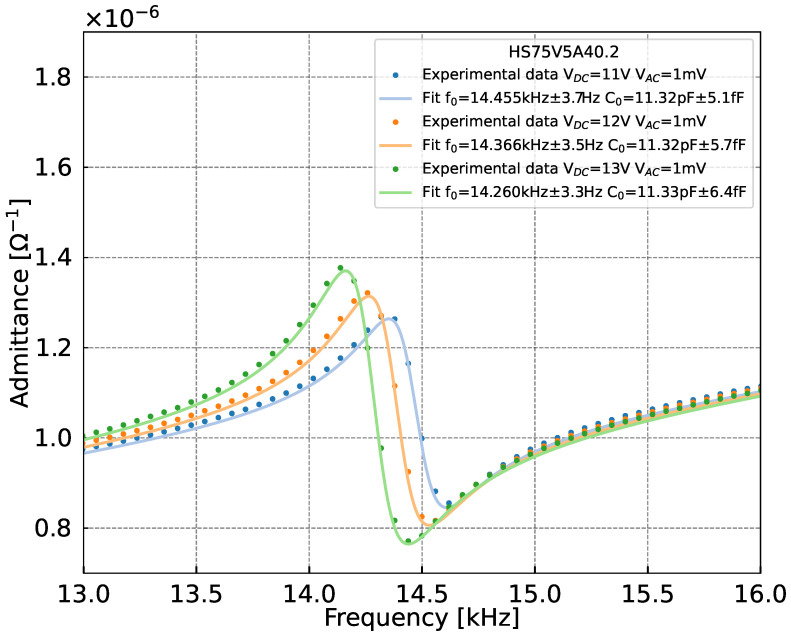
The electrical admittance variation as a function of the frequency for polarization voltages of 11, 12 and 13 V, using a 1 mV drive voltage. Points are the experimental data, and continuous lines are fitted curves derived from the Butterworth–Van Dyke model.

The frequency response of the MEMS is analyzed as a function of different DC bias voltages (Figure 5). Each response exhibits a main resonance peak, followed by an anti-resonance peak caused by the feedthrough capacitance $C_{0}$. Far from the resonance frequency, without any mechanical excitation, the MEMS remains at rest and the capacitance is essentially equal to $C_{0}$. In this region, the output signal is linear and its slope is proportional to $C_{0}$, following the equation:

(4) $$C_{0}=\frac{V_{out}}{G_{TA}\cdot V_{exc}\cdot 2\cdot \pi \cdot f}$$

where *f* is the drive voltage $V_{\mathrm{exc}}$ frequency.

The fitting was performed using the Butterworth-Van Dyke model [12] to estimate the resonance frequency, the quality factor and the capacitance $C_{0}$. The experimentally extracted feedthrough capacitance $C_{0}$ is estimated as 11.3 pF, while the theoretical value is calculated as 10.52 pF, corresponding to a relative error of approximately 7%. The value of $C_{0}$ should be maximized, as a larger capacitance indicates more efficient electro-mechanical conversion, resulting in a greater sensitivity of the global sensor. In our study, the feedthrough capacitance $C_{0}$ have two contributions. The dominant component is due to the 3 µm air gap between the arms and the substrate. The additional contributions originate from other structural elements of the resonator, such as square supports, creating a capacitor. The latter are named by parasitic capacitance $C_{p}$ and should be minimized. The output signal attenuation can be determined using the following ratio [13]:

(5) $$Attenuation=\frac{C_{0}}{C_{0}+C_{p}}$$

Using the theorical parasitic capacitance $C_{p}$ value (=7.97 pF), the obtained attenuation factor is 0.59, which is of the same order than that of the latest reported resonator (0.65) [7]. This result is consistent with expectations, since both devices share the same geometry except for the lateral clearance above the back opening, which was 50 µm in [7] compared to 40 µm in the present work. Resonance frequency changes are also observed on Figure 5, as the polarization voltage increases. This effect is caused by the electrostatic force induced by the applied DC bias, which modifies the effective mechanical stiffness of the MEMS structure. In addition to the frequency shift, the electrical signal amplitude also rises with increasing polarization, due to enhanced actuation efficiency. However, above the pull-in voltage, a collapse of the resonator will occur, as reported in [14]. To prevent this, an optimal polarization voltage of 13 V was selected for further measurements, leading to a resonance at 14.26 kHz and a quality factor of 51.

## 4. Photoacoustic Measurements

**Figure 6:**
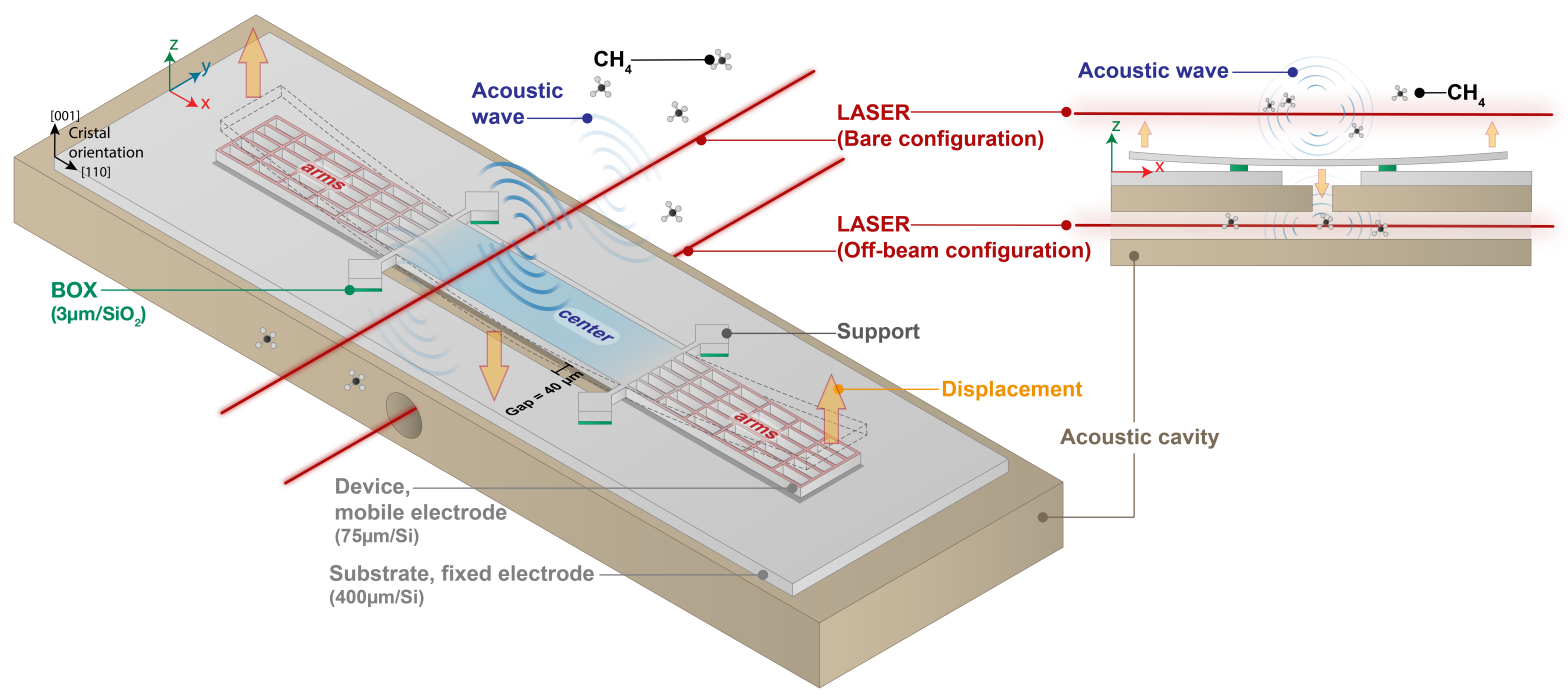
Two measurement configurations are studied. In the first one, referred to as the Bare configuration, the laser beam is directed above the MEMS. In the second one, called the Off-beam configuration, the laser beam passes through the acoustic cavity.

This section presents the sensor performance and compares the results obtained with and without the use of an acoustic cavity. To this end, two measurement configurations, illustrated in Figure 6, are investigated. The first configuration, referred to as the Bare configuration, consists of focusing the laser beam directly above the MEMS without involving the acoustic cavity. The second, called the Off-beam configuration, uses the acoustic cavity, with the laser beam passing through it.

**Figure 7:**
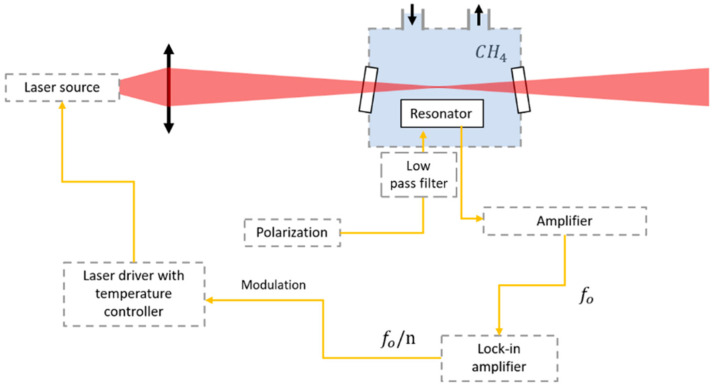
Illustration of the MEMS characterization setup for methane detection [10].

The experimental setup used for the MEMSPAS characterization is illustrated in Figure 7. The modulated laser used is a NORCADA distributed feedback (DFB) source emitting at 2.3 µm. According to the HITRAN database [15], this wavelength targets a strong and isolated methane absorption line at ν=4300.36 ${\mathrm{cm}}^{-1}$, with an absorption cross-section of $\sigma =3.7\times 10^{-21}$ ${\mathrm{cm}}^{2}$ ${\mathrm{molecule}}^{-1}$. The laser provides 3.9 mW output power at 140 mA and has a threshold current of 40 mA at 25 °C. It is driven by a Thorlabs ITC4002QCL LDITC controller (Thorlabs Inc., Newton, NJ, USA) and modulated at the MEMS resonance frequency $f_{0}$. The laser beam is focalized into an aluminium gas cell equipped with gas inlet and outlet ports and filled with methane diluted in nitrogen. The cell includes tilted ${\mathrm{CaF}}_{2}$ windows (20°) to prevent optical interferences and laser feedback. Inside the cell, the beam is directed either above the MEMS (Bare configuration) or through the acoustic cavity (Off-beam configuration). To generate a capacitive signal from its mechanical displacement, the device is polarized by a DC bias voltage $V_{\mathrm{DC}}$. The resulting signal is amplified by a FEMTO LCA-40K-100M transimpedance amplifier (FEMTO Messtechnik GmbH, Berlin, Germany) with a gain of $10^{8}$ V/A, and then demodulated at $f_{0}/n$ (with n=1 for 1*f*) using a MFLI lock-in amplifier. All measurements are conducted at room temperature and atmospheric pressure. Prior to each measurement, the gas cell is purged with dry nitrogen to eliminate all residual gases and ensure well-controlled ${\mathrm{CH}}_{4}$ concentrations.

### 4.1. Characterization

**Figure 8:**
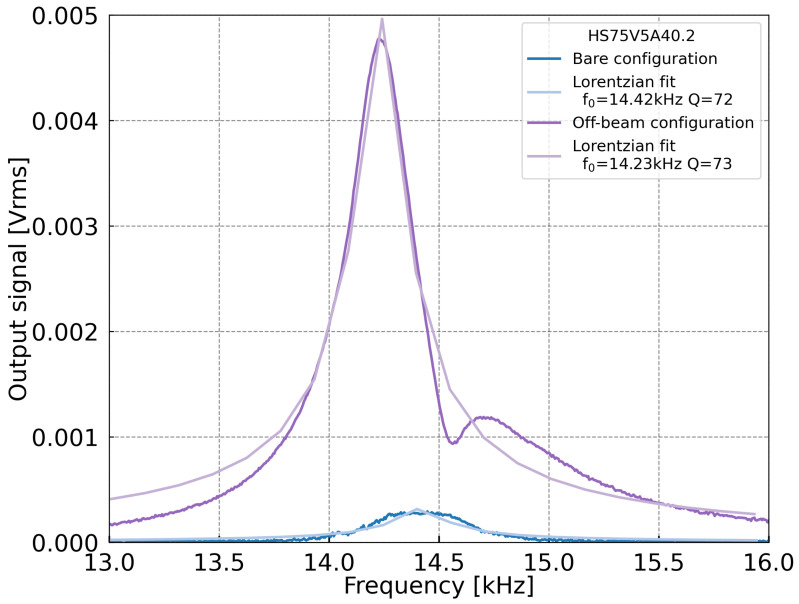
Frequency response of the H-squared resonator capacitive signal with 1% ${\mathrm{CH}}_{4}$ in the Bare configuration (blue curve) and Off-beam configuration (purple curve), using a polarization bias of 13 V. The resonance frequencies and quality factors are extracted from Lorentzian fits.

To characterize the MEMS, the methane absorption line at ν=4300.36 ${\mathrm{cm}}^{-1}$ was selected. For this purpose, the laser was set to 139 mA at 25 °C and modulated with a 9.6 mA amplitude, settings optimized for first-harmonic (1f) detection. As a first step, measurements were carried out with the MEMS alone, using a polarization bias of 13 V to maximize the signal and a ${\mathrm{CH}}_{4}$ concentration of 1%. A frequency sweep revealed a resonance at 14.42 kHz, with a quality factor of 72. Based on this result and using the analytical expressions provided in Section 2.2, the acoustic cavity dimensions were calculated to match the MEMS resonance frequency. Table 1 summarizes all the dimensions chosen. The MEMS was then mounted onto the 3D-printed acoustic cavity. Figure 8 shows the frequency response of the capacitive signal in both Bare and Off-beam configurations. A resonance at 14.42 kHz is observed in the Bare case, and a resonance at 14.23 kHz is seen in the Off-beam configuration. This frequency difference observed can be due to a mismatch between the acoustic cavity and the MEMS resonator frequencies, as the proximity of the MEMS to the branch tube perturbs the acoustic field and slightly shifts the cavity resonance. In addition, the Off-beam curve exhibits an additional resonance peak at a higher frequency, the origin of which is unclear at this stage. Nevertheless, the signal amplitudes measured in the Bare and Off-beam configurations are 0.29 mV and 4.8 mV, respectively. This confirms that the acoustic cavity significantly enhances the sensor response.

### 4.2. Photoacoustic Signal

**Figure 9:**
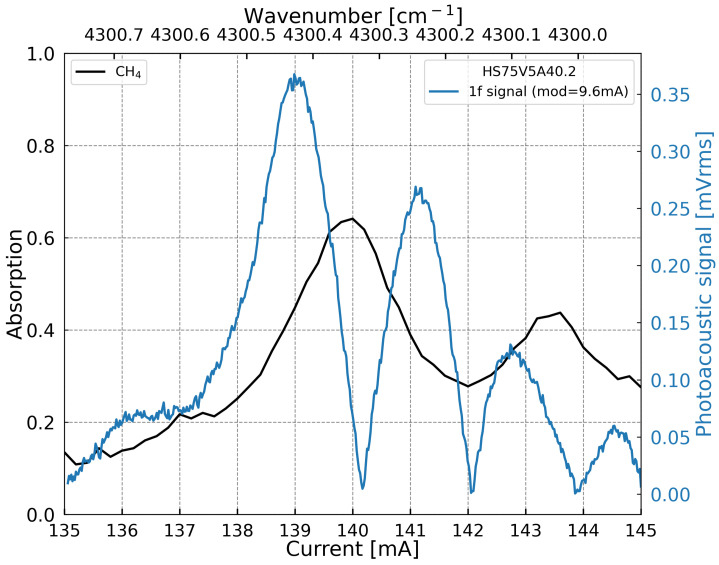
Absorption spectrum of 100% methane measured with a photodiode (black curve) over a 3 cm optical path, and the corresponding photoacoustic signal of the MEMS first harmonic (1f) response in the Off-beam configuration (blue curve), plotted as a function of the DFB laser injection current and the corresponding wavenumber. The photoacoustic measurement was performed at atmospheric pressure for a methane concentration of 800 ppmv. The DFB laser emits around 2.3 µm with an output power of 3.9 mW at an injection current of 140 mA.

The objective of this section is to verify the absence of any photothermal contribution in the measured signal, ensuring that the laser energy is absorbed exclusively by the target gas and not by the MEMS structure itself. A spectral scan is then performed by varying the laser current from 135 mA to 145 mA, corresponding to a slight wavelength shift from 2.3220 µm to 2.3216 µm, respectively. The laser beam is modulated at the MEMS resonance frequency (14.23 kHz), with a modulation amplitude of 9.6 mA. The gas cell is filled with 800 ppmv of ${\mathrm{CH}}_{4}$ diluted in pure nitrogen. First, a direct absorption measurement is carried out using a photodiode placed on the optical axis at the cell output, as a typical TDLS configuration. Then, the photoacoustic signal is recorded using the MEMS resonator, biased at 13 V, over the same current range. Figure 9 shows both measurement results. The 1f photoacoustic signal corresponds to the first derivative of the ${\mathrm{CH}}_{4}$ absorption peak. The maximum photoacoustic response is obtained at a laser current of 139 mA at 25 °C. These operating conditions are used for the following experiments.

### 4.3. Displacement Measurement

**Figure 10:**
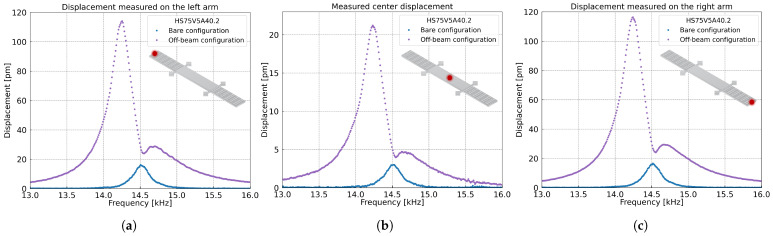
Displacement measured with a Laser Doppler Vibrometer (LDV) at the (**a**) left arm extremity, (**b**) center, and (**c**) right arm extremity of the MEMS. The measurements were performed with 1% ${\mathrm{CH}}_{4}$ concentration and a polarization voltage of 13 V.

The displacement of the MEMS was measured optically under photoacoustic excitation in both Bare and Off-beam configurations. A Polytec OVF-5000 Laser Doppler Vibrometer (Polytec GmbH, Waldbronn, Germany) with a 3 µm laser spot diameter was used, with a VD-06 decoder set at 2 mm/s/V. Figure 10 shows the MEMS displacement during a frequency sweep performed with 1% ${\mathrm{CH}}_{4}$ and a polarization voltage of 13 V. The displacement was measured at three locations: the left arm extremity, the center, and the right arm extremity. Measured displacements are in the picometer range. The frequency response profile closely matches the one obtained from capacitive measurements, as shown in Figure 8, confirming the enhanced signal in the Off-beam configuration. The displacement of the arms increases from 15 pm in Bare configuration to 115 pm in Off-beam. At the same time, the center of the MEMS exhibits limited motion, with a displacement of about 22 pm in Off-beam. The ratio between the arm and center displacements in Off-beam is then approximately ≈ 5.2. This low displacement at the center reduces the effective mass of the resonator, thereby decreasing its mechanical inertia. As a result, the arms can respond more efficiently to the acoustic excitation, maximizing their displacement and the overall output signal. The left and right arms exhibit similar displacements, confirming the symmetry and uniformity of the structure. This consistency reflects the good quality of the fabrication process, most likely due to the constant rotation of the sample during the HF vapor release step, which ensures a uniform etching and a balanced release of the resonator arms.

### 4.4. Linearity Measurements

**Figure 11:**
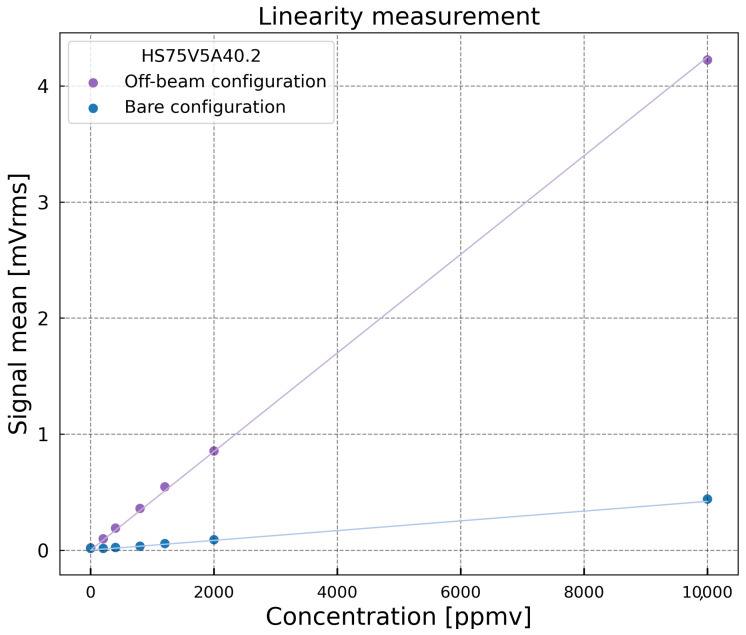
Output signal of the MEMS as a function of the injected methane concentration for the Off-beam and Bare configurations, fitted with a linear regression.

The evaluation of the sensor linearity is essential to assess the quality and reliability of its response. The 1*f* photoacoustic signal was measured for increasing methane concentrations, ranging from 200 ppmv to 10,000 ppmv, which were successively injected into the gas cell. A total of seven concentrations were tested. Each concentration was prepared using a mass flow controller Alytech GasMix Aiolos II (Alytech, Juvisy-sur-Orge, France), and the corresponding signal was recorded. Between each measurement, the cell was flushed with pure $N_{2}$ for 10 min to ensure full recovery of the baseline signal. Figure 11 shows the results of the linearity test for both Off-beam and Bare configurations. The 0 ppmv point was obtained with pure nitrogen, allowing the evaluation of any photothermal contribution in the absence of an absorbing species. The red linear fits confirm the linear behavior of the sensor over the tested concentration range.

### 4.5. Allan-Werle Deviation

**Figure 12:**
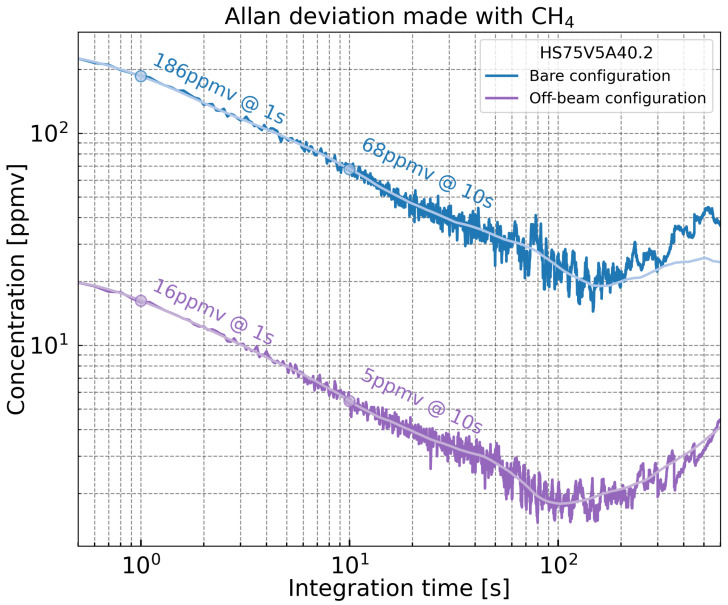
Allan-Werle deviation calculated from a 30 min acquisition for Bare and Off-beam configurations, with a ${\mathrm{CH}}_{4}$ concentration of 800 ppmv and a lock-in time constant of 100 ms.

To evaluate the stability and sensitivity of the gas sensor, the limit of detection (LOD), defined as the lowest measurable gas concentration, was assessed. An Allan-Werle deviation analysis was performed on the 1*f* photoacoustic signal recorded over a period of 30 min. The background signal, obtained from a measurement performed with no gas present, was subtracted in order to avoid including any photothermal component in the final measurement. The gas cell was filled with 800 ppmv of methane. The laser current was set to 139 mA with a 9.6 mA modulation amplitude and the time constant was fixed ar 100 ms. The MEMS was polarized with a 13 V bias voltage. Both Bare and Off-beam configurations were studied. Figure 12 presents the Allan-Werle deviation calculated, as a function of the integration time τ. For τ<1 s, the observed decrease is attributed to the low-pass filter of the lock-in amplifier. For τ between 1 s and 100 s, a slope proportional to $\tau^{-1/2}$ is observed, indicating the dominance of white noise and good short-term stability. Beyond τ = 400 s, long-term drift becomes significant. The LOD at 1 s of integration time is 186 ppmv in the Bare configuration and 16 ppmv in the Off-beam configuration. At 10 s, the LOD is 68 ppmv in Bare and remains 5 ppmv in Off-beam, showing the improved stability of the gas sensor. Its performance depends on the laser power, the gas absorption cross-section, and the detection bandwidth. A normalized quantity, the Normalized Noise-Equivalent Absorption (NNEA) metric, is used to compare sensor performances independently of these parameters. It is defined as

(6) $$NNEA=\frac{\alpha_{LOD}\cdot P}{\sqrt{\Delta f}}$$

where $\alpha_{LOD}$ is the absorption coefficient at the detection limit, *P* is the laser power, and Δf is the detection bandwidth. Lower NNEA values indicate better sensor performance. Using the measured LOD at 1 s, the NNEA is calculated to be $1.49\times 10^{-7}$ W·${\mathrm{cm}}^{-1}\cdot$ ${\mathrm{Hz}}^{-1/2}$ for the Bare configuration and $1.28\times 10^{-8}$ W·${\mathrm{cm}}^{-1}\cdot$ ${\mathrm{Hz}}^{-1/2}$ for the Off-beam configuration.

## 5. Discussion and Perspectives

This work aimed to develop a compact gas sensor combining high sensitivity and selectivity. To achieve this, a photoacoustic technique named MEMSPAS was developed, based on a resonant capacitive MEMS specifically designed for gas detection via photoacoustic spectroscopy. Unlike microphones, this MEMS offers a higher quality factor. It can be used with various gases, as long as the laser wavelength corresponds to an absorption band of the target species.

A previously optimized MEMS resonator design [7] was integrated with a T-shaped acoustic cavity tuned to its resonance frequency. This configuration focuses the photoacoustic pressure at the center of the structure, significantly enhancing excitation efficiency compared to the Bare setup, where waves are distributed along the optical axis.

The electrical characterization and the measurement of the photoacoustic signal enabled the identification of optimal operating parameters for the MEMS sensor. A bias voltage of 13 V and a laser current of 139 mA were selected based on signal optimization.

Under these conditions, a frequency sweep revealed a precise resonance at 14.42 kHz. Consequently, the acoustic cavity was fabricated with a main tube length of 14.5 mm to match this resonance. The MEMS was subsequently mounted on the cavity to form the Off-beam configuration. The frequency response obtained in the Off-beam configuration displayed an unexpected bifrequency behavior and a resonance shift, that can be due to a mismatch between the acoustic cavity and the MEMS resonator frequencies, as the MEMS perturbs the cavity resonance.

In terms of sensitivity, a detection limit of 16 ppmv at 1 s integration time was achieved in the Off-beam configuration. This corresponds to a Normalized Noise-Equivalent Absorption (NNEA) value of $1.28\times 10^{-8}\;W\cdot {\mathrm{cm}}^{-1}\cdot {\mathrm{Hz}}^{-1/2}$, representing an 11-fold improvement compared to the Bare configuration.

To provide context for this performance, Table 2 compares representative photoacoustic CH_4_ sensors. In this comparison, the cavity-enhanced MEMSPAS presented in this work outperforms QEPAS systems, as indicated by its lower NNEA. However, state-of-the-art cavity-based MPAS sensors still exhibit better sensitivity, but the results obtained in this work show that our approach is steadily approaching these performance levels. Finally, this work reports the first integration of an acoustic cavity on a MEMS-based PAS. Compared to our previous MEMSPAS design [7], this new configuration leads to a clear improvement in sensitivity.

Overall, the strong reduction in the detection limit from 186 ppmv to 16 ppmv confirms the effectiveness of coupling a MEMS transducer with an acoustic cavity for highly sensitive gas detection. Further improvements are still possible through the optimization of the cavity’s geometry.

Future work will focus on investigating the influence of the MEMS resonator on the acoustic cavity in order to better understand how its presence perturbs the acoustic field and affects the overall sensor performance. Another direction will involve replacing the current gap-varying capacitive transduction method with a surface-varying approach to reduce viscous damping from gas trapped between the MEMS and the substrate. This change could further enhance the mechanical response and sensitivity of the sensor.

## Figures and Tables

**Table 1:** Dimensions of the acoustic cavity used in our study.

Name	Abbreviation	Dimension
Main tube length	$L_{\mathrm{mR}}$	14.5 mm
Main tube radius	$R_{\mathrm{mR}}$	0.390 mm
Branch tube length	$t_{0}$	1 mm
Branch tube radius	$r_{0}$	0.226 mm
Distance MEMS-cavity	*d*	400 µm

**Table 2:** Comparison of different photoacoustic ${\mathrm{CH}}_{4}$ sensor techniques.

Reference	Technic	Acoustic Cavity	NNEA (W·${\mathrm{cm}}^{-1}$·${\mathrm{Hz}}^{-1/2}$)
Hu et al., 2020 [16]	QEPAS	Cylindric	$1.80\times 10^{-8}$
Rouxel, 2015 [17]	MPAS	Helmholtz	$4.30\times 10^{-9}$
Seoudi et al., 2023 [7]	MEMSPAS	None	$8.58\times 10^{-8}$
This work	MEMSPAS	T-shaped	$1.28\times 10^{-8}$

## Data Availability

The data presented in this study are available on request from the corresponding author.
